# Vault RNA1–1 riboregulates the autophagic function of p62 by binding to lysine 7 and arginine 21, both of which are critical for p62 oligomerization

**DOI:** 10.1261/rna.079129.122

**Published:** 2022-05

**Authors:** Magdalena Büscher, Rastislav Horos, Ina Huppertz, Kevin Haubrich, Nikolay Dobrev, Florence Baudin, Janosch Hennig, Matthias W. Hentze

**Affiliations:** 1 European Molecular Biology Laboratory, 69117 Heidelberg, Germany; 2 Collaboration for joint Ph.D. degree between EMBL and Heidelberg University, Faculty of Biosciences, Heidelberg, Germany

**Keywords:** riboregulation, vault RNA, p62, autophagy, small ncRNA

## Abstract

Cellular processes can be regulated at multiple levels, including transcriptional, post-transcriptional, and post-translational mechanisms. We have recently shown that the small, noncoding vault RNA1–1 negatively riboregulates p62 oligomerization in selective autophagy through direct interaction with the autophagic receptor. This function is highly specific for this Pol III transcript, but the determinants of this specificity and a mechanistic explanation of how vault RNA1–1 inhibits p62 oligomerization are lacking. Here, we combine biochemical and functional experiments to answer these questions. We show that the PB1 domain and adjacent linker region of p62 (aa 1–122) are necessary and sufficient for specific vault RNA1–1 binding, and we identify lysine 7 and arginine 21 as key hinges for p62 riboregulation. Chemical structure probing of vault RNA1–1 further reveals a central flexible loop within vault RNA1–1 that is required for the specific interaction with p62. Overall, our data provide molecular insight into how a small RNA riboregulates protein–protein interactions critical to the activation of specific autophagy.

## INTRODUCTION

Macroautophagy ensures the clearance of intracellular substrates ranging from single ubiquitinated proteins to large proteotoxic aggregates and defective organelles. The human autophagy receptor p62 (also known as SQSTM1, ZIP, or ORCA) guides selective macroautophagy of intracellular cargo to maintain homeostasis in situations of proteotoxic stress or starvation (Bjørkøy et al. 2005; Lim et al. 2015; Dikic 2017). Following activation, p62 oligomerizes and forms large protein assemblies—so-called sequestosomes—that guide intracellular cargo to the elongating autophagic membrane (Jakobi et al. 2020). Upon complete enclosure of the assemblies, the autophagosome fuses with lysosomes to degrade its content and recycle the building blocks for further cellular utilization.

In this process, p62 recognizes ubiquitinated cargo via its carboxy-terminal ubiquitin-associated (UBA) domain (Zaffagnini et al. 2018) and N-arginylated proteins via its ZZ-type zinc finger domain, respectively (Cha-Molstad et al. 2017). The central LC3-interacting region (LIR) binds the ATG8-like proteins LC3 and GABARAP, thereby bridging intracellular cargo to the autophagic membrane (Pankiv et al. 2007).

Since the affinity of p62 monomers for specific cargo and the ATG8-like proteins is rather modest, protein oligomerization is essential for achieving high avidity protein interactions while maintaining selectivity (Wurzer et al. 2015). Oligomerization is mediated by the amino-terminal Phox and Bem1 (PB1) type I/II domain that comprises an OPCA motif (short for OPR: octicosapeptide repeat, PC: Phox and Cdc motif, AID: Atypical protein kinase C interaction domain) and a conserved lysine which can align in a head to tail-fashion (Ito et al. 2001; Lamark et al. 2003; Ciuffa et al. 2015). Strikingly, mutations that disrupt PB1 domain-mediated oligomerization prevent p62 engagement in autophagy and thereby highlight the “effector” function of PB1-mediated oligomerization in this process (Lamark et al. 2003; Bjørkøy et al. 2005; Itakura and Mizushima 2011; Sun et al. 2018; Jakobi et al. 2020). Intrinsic modulators of p62 oligomerization include the formation of stabilizing disulfide bonds between cysteine 105 and 113 upon oxidative stress or ZZ domain binding (Cha-Molstad et al. 2017; Carroll et al. 2018), as well as inhibitory post-translational modifications within the oligomerization interface (Christian et al. 2014; Pan et al. 2016). Besides, the linker region between the PB1- and ZZ domain of p62 (aa 100–113) was shown to auto-regulate p62 by interaction with the ZZ domain (Zhang et al. 2018).

We recently discovered that the small noncoding vault RNA1–1 directly binds to p62 and regulates p62 oligomerization and hence function in autophagy (Horos et al. 2019; Büscher et al. 2020). Vault RNAs are transcribed by RNA polymerase III and were originally identified more than three decades ago as components of the so-called vault particle (Kedersha and Rome 1986). The four human vault RNA paralogs share almost identical sequences at their 3′ and 5′ ends while their central domains vary in sequence and length (Supplemental Fig. 3; Nandy et al. 2009; Stadler et al. 2009). We found that vault RNA1–1 is a prime p62-interacting vault RNA, but the molecular determinants of their specific interaction and the mechanism of how these control oligomerizations remained unresolved (Horos et al. 2019). We previously described a ZZ domain mutant (R139A/K141A) and a PB1 domain mutant (R21A/D69A/D73A) that both exhibit reduced RNA binding while showing increased or diminished oligomerization, respectively (Horos et al. 2019). These findings implicated a role for both domains in riboregulation, suggesting that either the PB1 domain itself or PB1 domain-mediated oligomerization may be required for p62's RNA binding activity (Horos et al. 2019). Here, we delineate key determinants of p62 and vault RNA1–1 that mediate binding and specificity of the p62/vault RNA1–1 interaction. We uncover the specific importance of the two amino acids lysine 7 and arginine 21 in riboregulation and suggest their function as hinges between oligomerization and RNA binding. We also identify the central loop of vault RNA1–1 as a specific determinant of p62 binding compared to the other human vault RNAs. Thereby our results elucidate critical molecular details of this prime example of riboregulation.

## RESULTS

### The PB1 oligomerization domain and adjacent linker region of p62 mediate specific vault RNA1–1 binding

**FIGURE 1.**
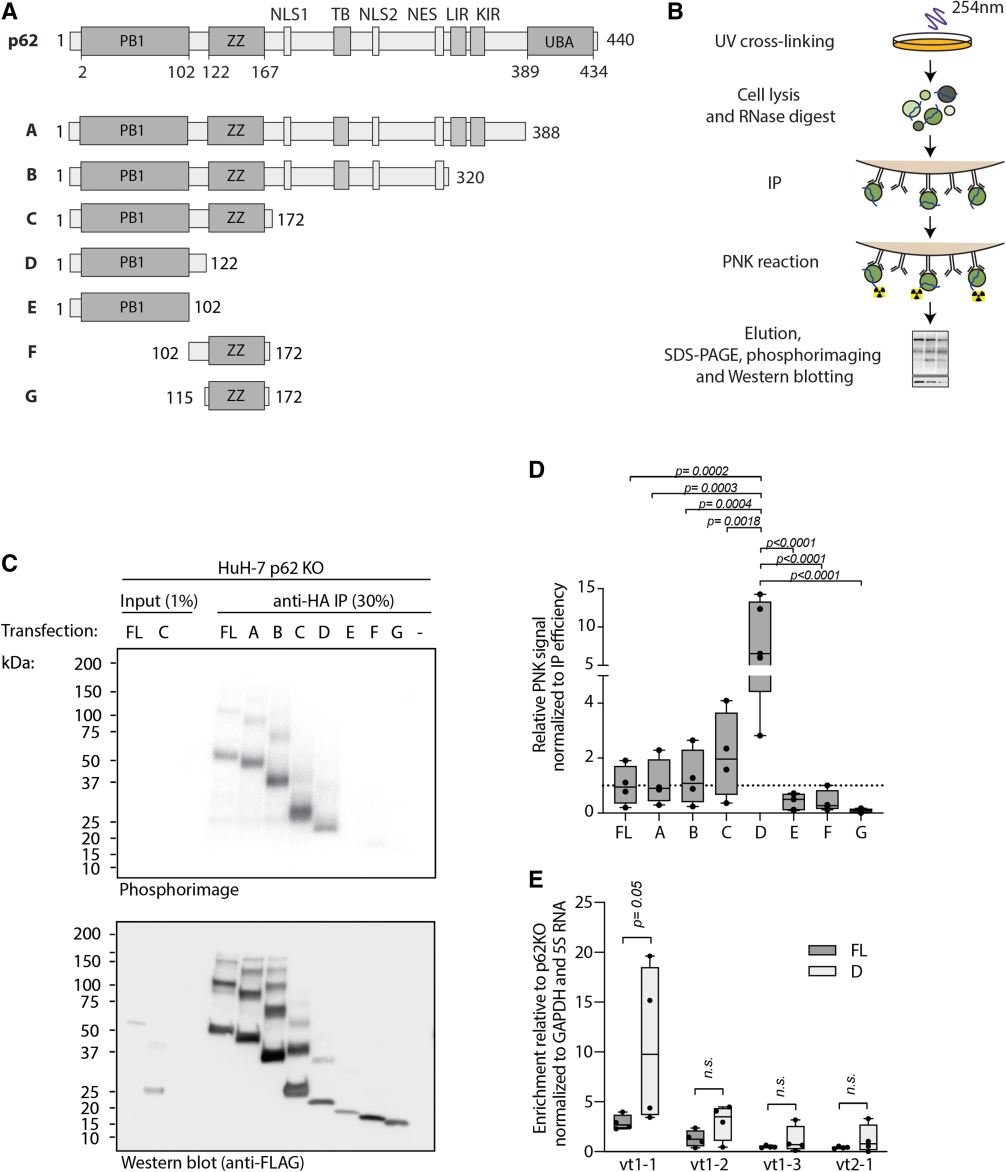
The PB1 domain and adjacent linker region are necessary and sufficient for p62's RNA binding capacity and specificity toward vault RNA1–1. (*A*) Schematic overview of full-length p62 and truncation constructs with amino-terminal FLAG-HA tags. (PB1) Phox and Bem1, (ZZ) ZZ-type zinc finger, (NLS) nuclear localization signal, (NES) nuclear export signal, (TB) TRAF binding region, (LIR) LC3 interacting region, (KIR) Keap interacting region, (UBA) ubiquitin associated domain.] (*B*) Schematic overview of T4 polynucleotide kinase labeling assay (PNK). Cells are UV-crosslinked to establish a covalent bond between proteins and RNA at zero-distance. Subsequently, lysates are treated with RNase A and used for immunoprecipitation followed by radioactive labeling of RNA with T4 polynucleotide kinase. (*C*) PNK. Full-length (FL) p62 or different p62 truncations were expressed by transient transfection in HuH-7 p62 knockout cells and RNA binding capacity determined as described in *B*. (*D*) Quantification of PNK assays as in *C*. Adjusted *P* values are indicated according to one-way ANOVA with Tukey correction for multiple comparisons (*n* = 4). (*E*) Native immunoprecipitation of FLAG-HA-p62 full-length (FL) or truncation D from transfected HuH-7 p62 KO cells followed by quantitative RT-PCR of bound RNA. Indicated are adjusted *P* values from unpaired *t*-tests with Holm–Sidak correction for multiple comparisons (*n* = 4) (vt1–1: vault RNA1–1).

To identify the region of p62 that is required for RNA binding in a systematic manner, we generated a series of FLAG-HA tagged p62 truncation constructs (Fig. 1A), and assessed their RNA-binding capacity via polynucleotide kinase (PNK) assay in p62 knockout (KO) HuH-7 cells (Fig. 1B). In this assay, UV-crosslinking introduces covalent bonds between p62 and bound RNAs. Subsequently the cells are lysed, and lysates subjected to limited RNase treatment before p62 immunoprecipitation and end-labeling of crosslinked RNA with PNK (Fig. 1B). The eluates are resolved by SDS-PAGE and total RNA binding is assessed by phosphorimaging and western blotting, respectively. Although UV-crosslinking is not considered to promote protein–protein crosslinking in general (Greenberg 1979; Pashev et al. 1991; Suchanek et al. 2005), we noticed earlier that p62 oligomers can be UV-crosslinked, allowing the assessment of the oligomerization status of p62 in addition to RNA binding (Horos et al. 2019).

We find that the amino-terminal 172 amino acids of p62 carry the full RNA-binding capacity compared to the full-length (FL) protein (Fig. 1C,D). Additional deletion of the ZZ domain, leaving only the PB1 domain and adjacent carboxy-terminal linker region of p62 (p62_1–122_; aa 1–122) significantly increases normalized RNA binding above the level of wild-type p62, suggesting a negative modulatory function of the ZZ domain on RNA binding. However, the PB1 domain alone (aa 1–102) or the ZZ domain with the linker (aa 102–172) display little if any RNA-binding capacity (Fig. 1C,D). These data map the relevant RNA-binding interfaces to p62_1–122_. In contrast, the ZZ domain (aa 115–172) may play a regulatory role (Fig. 1C,D; see below).

To specifically assess vault RNA1–1 binding under steady-state (i.e., noncrosslinking) conditions, we performed native immunoprecipitation followed by RT-qPCR of copurified RNA (RIP). In this assay, p62_1–122_ displays specific and maximal vault RNA1–1 binding compared to full-length p62 (Fig. 1E; Supplemental Fig. 1). In contrast, p62 association with the other vault RNA paralogs is not significantly changed. These findings reflect the prime role of vault RNA1–1 in p62 riboregulation as previously observed (Horos et al. 2019) and hint toward differences between the vault RNA paralogs that mediate differential binding.

We conclude that p62_1–122_—the PB1 oligomerization domain with its adjacent carboxy-terminal linker—accounts for specific vault RNA1–1 binding to p62. This result is unexpected in light of the previously identified ZZ domain mutant R139A/K141A, which showed reduced RNA binding and implicated the ZZ domain as a critical region for the RNA interaction (Horos et al. 2019). The data presented here shed new light on this mutant and suggest a regulatory role for the ZZ domain in RNA binding.

### Hinge residues K7 and R21 are necessary for both, RNA binding and p62 oligomerization

**FIGURE 2.**
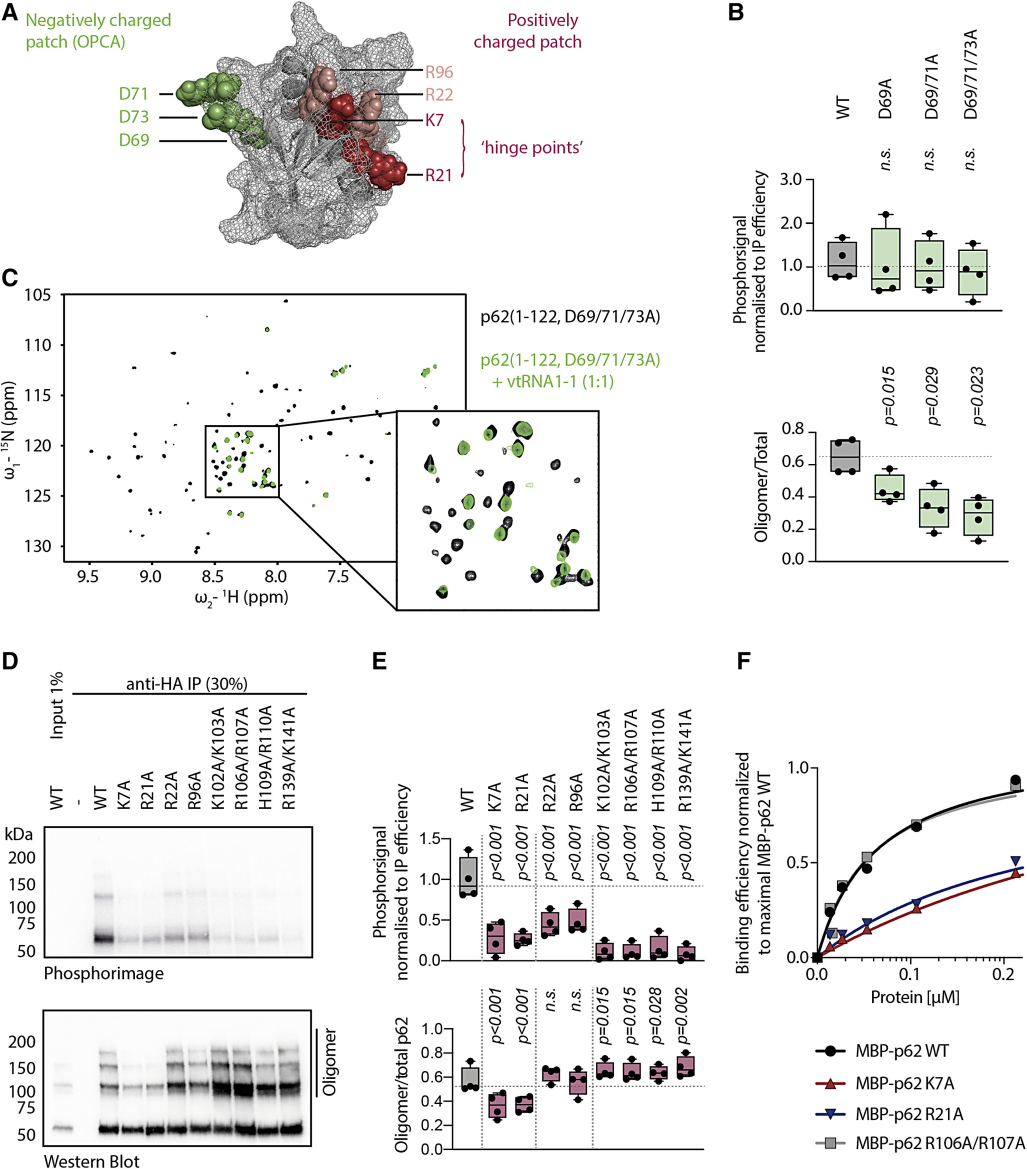
Lysine 7 and arginine 21 are hinge points for p62 riboregulation. (*A*) p62 PB1 domain structure (PDB ID: 2KKC). Negatively and positively charged residues are highlighted in green and red, respectively. (*B*) Quantification of radioactive signal and oligomerization in PNK assays of full-length FLAG-HA-p62 oligomerization mutants expressed in HuH-7 p62 KO cells. Significant differences from the WT construct were assessed by RM one-way ANOVA with Benjamini and Hochberg correction for multiple comparisons. (n.s.: not significant; *n* = 4). (*C*) ^1^H, ^15^N-HSQC NMR spectrum of p62(1–122, D69A/D71A/D73A) recorded with (green) and without (black) equimolar amounts of in vitro transcribed full-length vault RNA1–1. Most peaks show dramatic intensity loss upon RNA addition. Zoom-in highlights chemical shift perturbations for the remaining sharp peaks in the central region. (*D*) Representative PNK assay of full-length FLAG-HA-p62 RNA binding mutants expressed in HuH-7 p62 KO cells. (*E*) Quantification of radioactive signal and oligomerization in PNK assays of full-length FLAG-HA-p62 RNA binding mutants expressed in HuH-7 p62 KO cells. Significant differences from WT construct were assessed by RM one-way ANOVA with Benjamini and Hochberg correction for multiple comparisons. (n.s.: not significant; *n* = 4). (*F*) Quantification of representative EMSA with 10 nM radioactively labeled vault RNA1–1, 60 µM BSA, 150 nM bacterial tRNAs, and increasing amounts of recombinantly expressed and purified MBP-p62 WT, MBP-p62 K7A, MBP-p62 K21A, and MBP-p62 R106A/R107A.

We next generated FLAG-HA tagged mutant constructs of p62 to (i) assess, whether RNA binding depends on oligomerization, and (ii) to narrow down the RNA-binding interface in the context of full-length p62. To prohibit PB1-dependent oligomerization, we mutated the negatively charged OPCA motif (Fig. 2A) introducing single (D69A), double (D69A/D71A), or triple amino acid (D69A/D71A/D73A) exchanges (Lamark et al. 2003). We assessed multimerization of the different constructs in cellulo by measuring the ratio of p62 complexes with slower migration in SDS-PAGE over the total p62 amounts in PNK assays. As expected, all mutants display decreased oligomerization compared to the wild-type control and to each other with increasing mutations (Fig. 2B; Supplemental Fig. 2B). Yet, their RNA-binding capacity is not significantly changed (Fig. 2B; Supplemental Fig. 2). In addition to the in cellulo experiments, we used maltose-binding protein (MBP)-tagging to generate recombinant p62 with low oligomerization potential (Reuten et al. 2016; Horos et al. 2019; Tarafder et al. 2019) and tested in vitro binding of vault RNA1–1. This analysis confirms that the RNA-binding affinity of p62 is not affected by a double mutation of residues D69 and D73 (Supplemental Fig. 2A).

Next, we performed ^1^H, ^15^N-HSQC NMR spectroscopy with the minimal construct p62_1–122_ which we forced into a monomeric form by the before-characterized triple mutation (D69A/D71A/D73A). We recorded spectra with and without equimolar amounts of in vitro transcribed full-length vault RNA1–1 to assess binding (Fig. 2C). The spectra revealed strong signal loss for most peaks and multiple chemical shift perturbations for the remaining peaks upon addition of the RNA, indicating complex formation. Together, these findings show that the PB1 domain and adjacent carboxy-terminal linker region of p62 are sufficient for direct and specific vault RNA1–1 binding and that oligomerization is not required for this interaction.

To pinpoint the RNA-binding interface of p62, we mutated positively charged amino acids within the PB1 domain (Fig. 2A) and adjacent linker region. This strategy yielded three classes of mutants (Fig. 2D,E). First, PB1 domain mutants with decreased RNA binding and impaired oligomerization—namely p62 K7A and K21A. Second, those that display decreased RNA binding without significant changes in oligomerization, including R22A and R96A. And finally, the linker mutants K102A/K103A, R106A/R107A, and H109A/R110A that show reduced RNA binding and increased multimerization. This latter group also includes the previously identified p62 ZZ domain mutant R139A/K141A (Horos et al. 2019), which we included for reference.

Previous mutational studies showed that residues K7 and R21 are necessary for p62 multimerization, while R22 is not (Lamark et al. 2003). The PNK assay reflects this finding, confirming its utility as a tool to monitor p62 multimerization. Our data highlight residues K7 and R21 as critical residues for RNA binding and hence riboregulation, suggesting that RNA binding directly interferes with multimerization.

In contrast, R22 and R96 appear to extend the RNA-binding interface without making a strong contribution to multimerization in the absence of RNA binding. Mutations in the linker region or ZZ domain of p62 both resulted in reduced RNA binding and increased presence of multimers (Fig. 2D,E). To distinguish whether in these cases the loss of RNA binding resulted in multimerization or conversely, multimer formation displaced the RNA, we performed EMSAs with recombinant p62 that we forced into a low-oligomeric form by MBP-tagging (Reuten et al. 2016; Horos et al. 2019; Tarafder et al. 2019). We observed decreased vault RNA1–1 association for recombinant MBP-p62 K7A and R21A (Fig. 2F; Supplemental Fig. 2C,D), confirming the direct involvement of these residues in RNA binding. In contrast, the RNA-binding capacity of the linker mutant MBP-p62 R106A/R107A (Fig. 2F) or the ZZ domain mutant MBP-p62 R139A/K141A was not impaired (Supplemental Fig. 2E). These results favor the interpretation that mutations targeting the linker and ZZ domain foster multimeric forms of p62 in cellulo that exclude RNA from binding to the autophagic receptor.

### Vault RNA1–1 forms a central flexible loop that mediates binding and specificity of the p62 interaction

**FIGURE 3.**
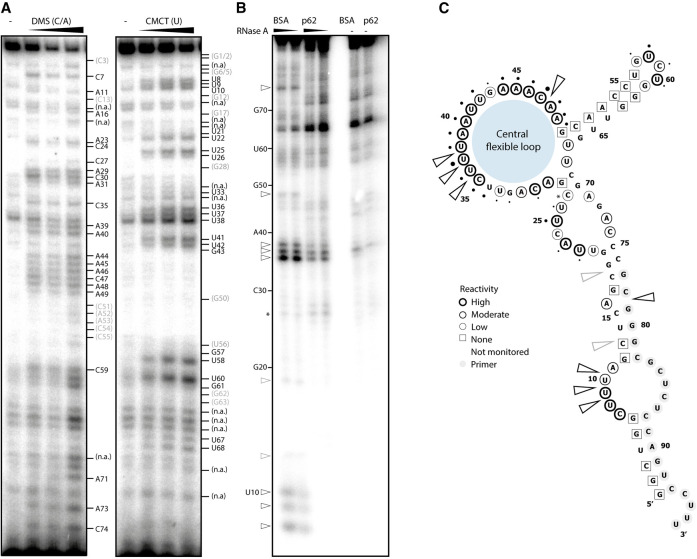
p62 interacts with a flexible loop in the central domain of vault RNA1–1. (*A*) Chemical structure probing of in vitro transcribed vault RNA1–1 in solution using Dimethyl sulfide (DMS) and 1-Cylcohexyl-(2-Morpholinoethyl)Carbodiimide metho-p-Toluene sulfonate (CMCT). The modified RNA is used for reverse transcription with a 5′-end labeled primer. The resulting cDNA is resolved on a denaturing urea-polyacrylamide gel. Residues with high and moderate reactivity are indicated in black and gray, respectively. Nonreactive residues are found in brackets. N.a. indicates that the reactivity is already observed for the nontreated control. (*B*) RNase A footprinting assay. In vitro transcribed vault RNA1–1 was radiolabeled at the 5′-end, incubated with recombinant MBP-p62 or BSA and subjected to limited RNase A digest followed by precipitation. RNase protection patterns were resolved on a denaturing urea-polyacrylamide gel. Arrows indicate RNase A protected nucleotides. (*C*) Secondary structure model of vault RNA1–1 based on chemical structure probing (circles corresponding to legend) with integration of RNase protection sites (arrows) from *B* and mean crosslink site values of vault RNA1–1 from p62-iCLIP analysis (dots). (The iCLIP data integrated in this figure were adapted from Horos et al. 2019.)

The tertiary structure of vault RNA1–1 is presently unknown. Previous secondary structure analyses using RNase H probing were useful but limited in resolution (Poderycki et al. 2005; Nandy et al. 2009). We applied chemical structure probing in solution to identify relevant features of vault RNA1–1 at nucleotide resolution (Fig. 3A,C, circles). The low reactivity of the conserved ends of vault RNA1–1 toward the probing reagents and their high base complementarity suggest that the ends of vault RNA1–1 form a base-paired stem. This interpretation fits well with previous thermodynamic models and is furthermore supported by the inter-species conservation of their base-pairing potential. In contrast, the central part of vault RNA1–1 forms a highly reactive flexible loop region that was previously not anticipated (Fig. 3A,C, circles). This finding is further supported by orthogonal transcriptome-wide RNA secondary structure probing data that are publicly available (Supplemental Fig. 4; Sun et al. 2019; Li et al. 2021).

Next, we conducted RNase A footprinting to identify vault RNA1–1 residues that are protected by p62 binding (Fig. 3B,C, gray arrows). Several protected regions emerge in the presence of p62. These include single-stranded bulges within the stem and, prominently, the central flexible loop. Of note, the protected nucleotides in the central loop completely match the crosslinked nucleotides previously identified by iCLIP of p62 (Fig. 3C, dots, Horos et al. 2019). When compared with the other three human vault RNA paralogs, the central loop of vault RNA1–1 differs considerably in length and sequence (Supplemental Fig. 3). Overall, our data implicate nucleotides within the central loop of vault RNA1–1 as specific p62 contact points.

### Nucleotides within the central flexible loop of vault RNA1–1 determine p62 riboregulation in cellulo

**FIGURE 4.**
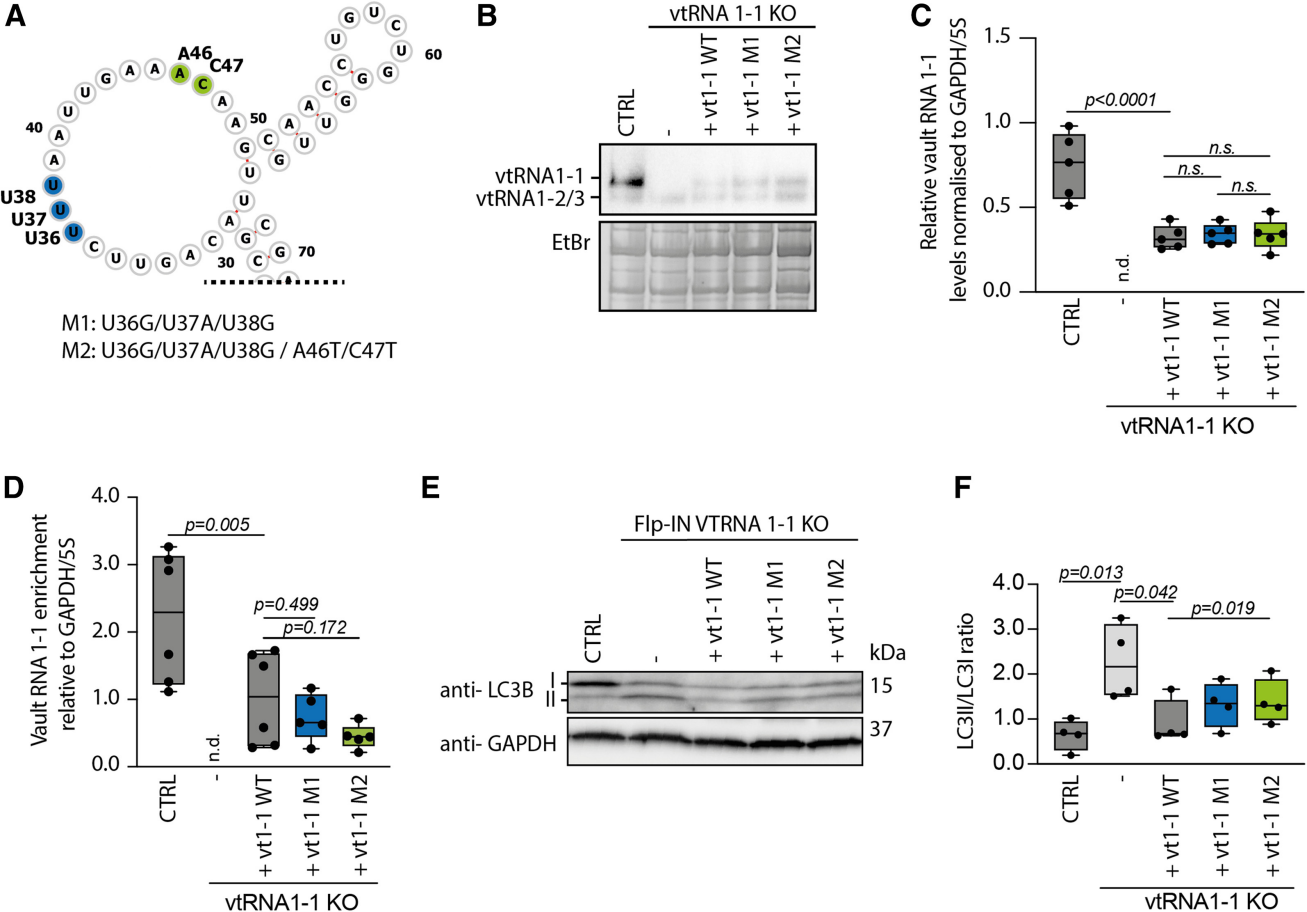
The flexible loop region is a key determinant of p62 riboregulation in cellulo. (*A*) Schematic representation of the flexible loop region. Sites of respective mutations are indicated by blue and green shading. (*B*) Northern blot analysis of HuH-7 Flp-IN cell lines that were used to stably express vault RNA1–1 and mutants thereof in a vault RNA1–1 KO background (please see Supplemental Fig. 5A). Vault RNA1–1, 1–2, and 1–3 were detected with a vault RNA specific, mutation-independent probe. (*C*) RT-qPCR. Expression of vault RNA1–1 and mutants thereof were detected with a specific, mutation-independent primer pair. Individual *P* values are indicated according to one-way ANOVA with Benjamini and Hochberg correction for multiple comparisons (*n* = 5). (*D*) Native p62 RIP followed by RT-qPCR. Vault RNA1–1 and mutants thereof were detected with specific, mutation-independent primer pair. Individual *P* values are indicated according to one-way ANOVA with Benjamini and Hochberg correction for multiple comparisons (*n* ≥ 5). (*E*) Western blot analysis of LC3B ratio. The cell lines were treated with 10 µM XIE62-1004-A for 4 h to induce p62-specific autophagy. Lysates were analyzed by western blot. (*F*) Quantification of LC3BII/I ratio in western blot analysis as performed in *E*. Individual *P* values are indicated according to RM one-way ANOVA with Benjamini and Hochberg correction for multiple comparisons. Significance was assessed by paired Student's *t*-test (*n* = 4).

To validate the functional importance of the central flexible loop region of vault RNA1–1 for riboregulation of p62, we generated a HuH-7 Flp-IN vault RNA1–1 KO cell line with a single FRT integration site. This KO cell line thus allowed the stable and isogenic reintegration of vault RNA1–1 variants expressed from the endogenous promoter (Supplemental Fig. 5). We compared reintegrated wild-type vault RNA1–1 with two mutants that alter either the central loop triplet U36/U37/U38 alone (M1) or in combination with residues A46/C47 (M2; Fig. 4A). The expression levels of all three reintegrated vault RNA1–1 variants are similar to each other and correspond to ∼50% of the vault RNA1–1 levels in the parental wild-type HuH-7 Flp-IN cell line (Fig. 4B,C). This expression level is in line with the single FRT integration site and, also reflected in a reduced vault RNA1–1 association with p62 as measured by native IP of p62 followed by RT-qPCR quantification (Fig. 4D; Supplemental Fig. 5E). As expected, the central domain mutants M1 and M2 tend to show reduced p62 binding in cellulo compared to the wild-type vault RNA1–1 (Fig. 4D; Supplemental Fig. 5E). This conclusion is further supported in vitro by EMSAs, where the mutations in the central loop of vault RNA1–1 compete less well for p62 binding than the wt counterpart (Supplemental Fig. 6A). This assay also shows that the central loop region of vault RNA1–1 alone is not sufficient for p62 binding in vitro (Supplemental Fig. 6A) and reveals the importance of the 3D structure of vault RNA1–1 for p62 binding.

Finally, we tested how the loop mutations affect p62 riboregulation in cellulo. As established before [19], we stimulated autophagy in the reconstituted cells with the synthetic, p62-specific ZZ-domain ligand XIE62-1004-A (Cha-Molstad et al. 2017) and assessed the LC3II/LC3I ratio as a measure of autophagic flux by western blotting. Consistent with our earlier data, the autophagic flux is significantly greater in cells depleted of vault RNA1–1 compared to the respective CRISPR/Cas9 control cell line upon treatment, as evidenced by the increased ratio of LC3BII/LC3BI (Fig. 4E,F). Reintegration of wild-type vault RNA1–1 rescues this phenotype significantly, whereas the central loop mutants fail to do so (Fig. 4E,F). Thus, residues within the central loop of vault RNA1–1 are required for the efficient riboregulation of p62-mediated autophagy in cellulo.

## DISCUSSION

Earlier work unveiled riboregulation as a new modality of post-translational protein regulation: to limit autophagic flux, p62 oligomerization is inhibited by direct vault RNA1–1 binding (Horos et al. 2019; Büscher et al. 2020). This discovery raised the key questions of how vault RNA1–1 interferes with oligomerization and how specificity is achieved compared to the other three human vault RNA paralogs and other tRNA-like Pol III transcripts. The work presented here addresses these mechanistic questions and uncovers critical features that determine binding and specificity of the p62/vault RNA1–1 riboregulatory pair. It reveals molecular details of how vault RNA1–1 modulates p62 function by interference with PB1 domain-mediated oligomerization. This work therefore explores an unprecedented function of noncoding RNA as post-translational protein regulators and defines critical mechanistic details of how vault RNA1–1 inhibits p62 oligomerization.

In detail, we show that the PB1 domain and adjacent linker region (p62_1–122_) are both necessary and sufficient for maximal and specific vault RNA1–1 binding. Mutational analyses pinpoint to K7 and R21 as critical amino acids that are necessary for RNA binding (Fig. 2) in addition to their previously known role in p62 oligomerization (Lamark et al. 2003). This result identifies these two residues as hinges for riboregulation, through which vault RNA1–1 inhibits p62 oligomerization and consequently autophagy.

Our initial report identified two mutants in the ZZ domain (K141A and R139A/K141A) that showed strongly diminished RNA binding and increased p62 oligomerization (Horos et al. 2019; see also Fig. 2D,E). Interestingly, the three new linker domain mutants K102A/K103A, R106A/R107A, and H109A/R110A display the same phenotype (Fig. 2E,F). Importantly, the ZZ domain had previously been shown to bind arginylated substrates (N-degrons) (Cha-Molstad et al. 2017; Zhang et al. 2018), a process that triggers p62 oligomerization and thereby initiates autophagic clearance. In this context, the linker region (p62_100–113_) was shown to exert negative auto-regulation through direct interaction with the ZZ domain (Zhang et al. 2018). In light of our new data (Fig. 2), we hypothesize that the above mutations within the linker region or the ZZ domain, respectively, render this auto-inhibitory mechanism dysfunctional, resulting in constitutive activation of the clearance pathway and increased p62 oligomerization. Of note, the auto-regulatory linker overlaps exactly with all PB1 domain mutants that show a significant increase in multimerization. In further support of this model, R139 has been shown to directly interact with the inhibitory linker and stabilize the auto-regulatory interaction (Zhang et al. 2018). The exclusion of RNA from p62 oligomers that are formed upon activation of ZZ domain-mediated autophagic clearance would ensure efficient aggregate clearance even in situations when cellular levels of the vault RNA1–1 riboregulator are high, including for example some viral infections (Mrázek et al. 2007; Nandy et al. 2009; Amort et al. 2015; Li et al. 2015).

As proposed before, our new data further corroborate that vault RNA1–1 primarily inhibits p62 oligomerization. Our new data also suggest that vault RNA1–1 may not be able to disrupt p62 oligomers that are formed when cargo binds to the ZZ domain. Integrating all available experimental evidence, we now suggest a refined mechanistic model for riboregulation of p62 by vault RNA1–1: under physiological, nutrient-replete conditions, vault RNA1–1 inhibits p62 oligomerization by binding the critical hinge points K7 and R21. When starvation reduces cellular vault RNA1–1 levels, p62 oligomerization via K7 and R21 is facilitated, stimulating autophagic flux (Supplemental Fig. 6B; Horos et al. 2019). In contrast, cargo binding to the ZZ domain and linker region during proteotoxic stress (Cha-Molstad et al. 2018; Zhang et al. 2018) triggers “sequestosome” formation and cargo clearance even when vault RNA1–1 levels are high, excluding vault RNA1–1 sterically in a dominant fashion (Supplemental Fig. 6B).

To also decipher the riboregulatory interface(s) of vault RNA1–1, we identified nucleotides required for p62 binding (Figs. 3, 4). We identify nucleotides within a central loop region of vault RNA1–1 that are necessary for the interaction and for specific riboregulation of p62 function in autophagy. However, further nucleotides likely contribute to an extended binding interface. Interestingly, this region of vault RNA1–1, especially nucleotides 45–50, had previously been shown to mediate apoptosis resistance in Hela cells (Amort et al. 2015; Bracher et al. 2020). While the molecular details of this phenotype remain unclear, our findings suggest the modulation of autophagy-dependent apoptosis via p62 as a possibility deserving of further exploration.

The p62/vault RNA1–1 binding interface may well be targeted by regulatory modifications that potentially influence riboregulation. For example, K7 has previously been found to be ubiquitinated by TRIM21 (Pan et al. 2016). Like vault RNA1–1 binding, this ubiquitination prevents p62 oligomerization and also facilitates proteasomal degradation of p62. In contrast, vault RNA1–1 riboregulation may represent a more dynamic and reversible form of autophagy inhibition that preserves p62 integrity. It will be interesting to systematically examine other protein or RNA modifications that could play a role for riboregulation.

Our data also demonstrate that autophagic clearance can be modulated by experimental changes of intracellular vault RNA1–1 levels (Fig. 4). Modulation of riboregulation could be especially beneficial in instances where the cargo load exceeds the capacity of cellular degradation mechanisms, as is the case in neurodegenerative diseases or cancer (Nixon 2013; Yang and Klionsky 2020).

In addition to its function as an autophagy receptor, p62 provides a platform for key cellular signaling pathways (Lee et al. 2010; Duran et al. 2011; Katsuragi et al. 2015; Moscat et al. 2016). While we have not detected changes in mTOR signaling upon depletion of vault RNA1–1 in HuH-7 cells (Horos et al. 2019), it will be interesting to explore globally whether RNA1–1 depletion or the expression of RNA-binding deficient p62 mutants affect other cellular signaling pathways.

With hundreds of newly identified RNA-binding proteins (Gebauer et al. 2020), riboregulation may represent a more widespread mechanism for the regulation of other key cellular processes beyond autophagy. From this perspective, the p62/vault RNA1-1 interaction offers a paradigm for the interference of a riboregulatory RNA with protein–protein interactions.

### Limitations of this study

This work provides a structure-function analysis to identify critical residues of p62 and vault RNA1–1, respectively, for the specific interaction between the two partners and their function in the riboregulation of p62 in mammalian autophagy. As such, it answers what distinguishes vault RNA1–1 from the other three human vtRNAs, and how vault RNA1–1 achieves control of p62 oligomerization via K7 and R21. In the future, it will be important to examine this interaction at atomic resolution using state of the art structural technologies.

Since our experiments have been conducted with cultured human cells, it will also be interesting to examine in vivo models (e.g., mice) expressing the mutant RNAs and forms of p62, respectively, which we have defined in this work. The investigation of animal models and clinical samples in the context of viral infections, cancer or neurodegenerative disorders will advance our understanding of the functional importance of p62 riboregulation in organismal function and disease.

## MATERIALS AND METHODS

### Antibodies and reagents

Antibodies and reagents used in this study are listed in Supplemental Table 1.

### Cell lines and culture conditions

HuH-7 cells (derived from a hepatocellular carcinoma of a human aged 57 yr) and their derivatives were cultured in DMEM containing 1 g/L glucose supplemented with 10% heat-inactivated FCS (Fetal Calf Serum, Gibco, Cat#: 10270-106), 2 mM l-glutamine (Thermo Scientific, Cat#: 25030081), and 100 U/mL penicillin/streptavidin (Thermo Scientific, Cat#: 15140122) at 37°C and 5% CO_2_. The cells were routinely passaged 2–3 times per week. For this purpose, the cells were dissociated through the addition of Trypsin-EDTA (0.05%, Gibco, Cat#: 25300-054). HuH-7 Flp-In cell line stocks and their derivatives were cultured in medium containing zeocin (100 µg/mL, InvivoGen, Cat#: ant-zn-05) or Hygromycin B Gold (200 µg/mL, InvivoGen, Cat#: ant-hg-1) depending on expressed marker genes. The parental hepatocellular carcinoma HuH-7 Flp-In cell line (one FRT integration site; Clone C2111) was first established in Beckmann et al. (2015), the HuH-7 p62 KO cell line in Horos et al. (2019).

### Cloning

Restriction-free cloning was performed according to van den Ent and Löwe (2006).

### CRISPR/Cas9 genome editing of cell lines

CRISPR/Cas9 genome editing was based on Cong et al. (2013) and Ran et al. (2013). In brief, guide RNAs targeting the vault RNA1–1 locus (Supplemental Table 2; Horos et al. 2019) were predicted using the CRISPOR online tool (http://crispor.tefor.net; Version May 2017), the highest-scoring candidates ordered from Sigma-Aldrich, annealed, and ligated into BbsI linearized pSpCas9(BB)-2A-GFP/RFP/Cer constructs (kindly provided by the Noh Laboratory, EMBL Heidelberg). Combinations of the generated plasmids were nucleofected into HuH-7 Flp-In cells using the SF Cell Line 4D-Nucleofector X Kit according to the manufacturer's guidelines, with 1Mio cells and 1 µg of total plasmid DNA in a 100 µL setup running program FF137. As a negative control, a mixture of all parental plasmids was used. A fluorescence-activated single-cell sort of double/triple-positive cells was performed 48 h after nucleofection. Upon expansion, the clones were tested for vault RNA1–1 deletion by PCR from genomic DNA using locus spanning primers (Supplemental Table 2). Cell lines lost the transiently transfected plasmids after outgrowth as checked via FACS.

### Transfections and treatments

Transfections were performed using Lipofectamine 3000 according to the manufacturer's guidelines. For cell-based assays, XIE62-1004-A (50 mM in DMSO; Horos et al. 2019), was diluted in PBS to a concentration of 2.5 mM and added to the medium in a final concentration of 10 µM.

### Native immunoprecipitation (IP)

For IPs, one confluent ∅︀ 15 cm dish per IP served as starting material. The cells were washed twice with ice-cold PBS. The buffer was aspirated completely and the cells lysed on ice through the addition of 750 µL Trit-Lysis buffer (20 mM Tris-HCl pH 7.4, 150 mM NaCl, 1 mM EDTA, 1 mM EGTA, 1% Triton X-100) supplemented with cOmplete Protease Inhibitor Tablets (Roche, Cat#: 11873580001) and 5 µg/mL RNasin (Promega, Cat#: N2511). The cells were collected through scraping and homogenized by pipetting. Lysates were cleared by centrifugation for 10 min at 13,000*g*, 4°C. The supernatant was transferred into a DNA LoBind 1.5 mL reaction tube (Eppendorf), the protein concentration determined by Bradford assay and the input material adjusted accordingly (1–2 mg/IP). Per IP, 25 µL anti-HA magnetic bead slurry was washed twice with PBS prior to addition to the samples. The IPs were performed for 1–2 h at 4°C with constant rotation. Following, the samples were washed six times with 1 mL of Trit-Lysis buffer and the reaction tubes exchanged after every second wash. For pH elution, 50 µL of 0.1 M glycine pH 2 were added per condition and incubated for 5 min at room temperature. The eluates were transferred into a new reaction tube and neutralized through addition of 7.5 µL of 1 M Tris-HCl pH 8.5.

For protein analysis, 1% of input material and 15%–20% of elution were analyzed by western blotting. For RNA analysis, 5% of input material and 70% of elution were processed by the addition of 400 µL RNA lysis buffer (Zymo) and RNA extraction using the Zymo Quick-RNA MicroPrep RNA Extraction Kit (Zymo), following the manufacturer's guidelines, including the DNase I on-column DNA digest. Elution was performed with 15 µL RNase-free H_2_O.

### Polynucleotide kinase (PNK) assay of FLAG-HA tagged proteins

For the PNKs, one 80%–90% confluent ∅︀10 cm or ∅︀15 cm dish per condition served as starting material. The cells were washed twice with ice-cold PBS, the PBS was aspirated completely, and the cells were UV-crosslinked at 150 mJ/cm^2^. Subsequently, the cells were lysed on ice with 0.75–1 mL PNK lysis buffer (50 mM Tris-HCl pH 7.4, 100 mM NaCl, 0.1% SDS, 1 mM MgCl_2_, 0.1 mM CaCl_2_, 1% NP40, 0.5% sodium deoxycholate supplemented with cOmplete Protease Inhibitors), and transferred into 1.5 mL reaction tubes. The lysates were homogenized by sonication on ice (Branson Cell Disruptor B15: 3 × 10 sec, 50% amplitude, level 4) and cleared by centrifugation at 16,000*g* for 10 min, 4°C. The protein concentration was measured by Bradford assay and the input material adjusted accordingly (1–2 mg/condition). Following, the lysates were treated with 5 ng/µL RNase A and 2 U/mL Turbo DNase for 15 min at 37°C and 1100 rpm. Per condition, 25 µL anti-HA magnetic bead slurry was washed twice with PBS prior to addition to the samples. The IPs incubated for 1.5–2 h at 4°C under constant rotation. Subsequently, the IPs were washed three times with PNK lysis buffer and three times with PNK wash buffer (50 mM Tris-HCl pH 7.4, 50 mM NaCl, 10 mM MgCl_2_, 0.5% NP-40, supplemented with cOmplete Protease Inhibitors). Radioactive labeling of retained RNA was performed on beads in PNK wash buffer containing 0.1 µCi/µL [γ-^32^P] ATP, 1 U/µL T4 PNK, and 1 mM DTT for 15 min at 37°C and 850 rpm. After another four washes with PNK wash buffer, the proteins were eluted by addition of 50 µL 0.1 M glycin pH 2 for 5 min and eluates neutralized with 7.5 µL 1 M Tris-HCl pH 8.5. The samples were complemented with 4× sample buffer containing 200 mM DTT, heated to 70°C for 3 min, resolved by SDS-PAGE and blotted onto nitrocellulose membranes. The membranes were rinsed, dried, and a radioactive signal was detected with a phosphorimaging screen for 3–72 h. Following, the membrane was used for western blot analysis. Image analysis was performed with Fiji Image J 2.0.0 (Schindelin et al. 2012, https://imagej.net/Fiji). The relative PNK signal was calculated and normalized to the relative western blot signal to account for variation in IP efficiency.

### SDS-PAGE and western blotting

For standard protein analysis, cells were washed twice with ice-cold PBS, the buffer aspirated completely and cells lysed through the addition of RIPA lysis buffer (25 mM Tris-HCl pH 7.6, 150 mM NaCl, 1% NP-40, 1% sodium deoxycholate, 0.1% SDS supplemented with cOmplete Protease Inhibitor and 0.1 U Benzonase/mL[Merck; Cat#71206]). Following, the cells were collected by scraping and protein concentration measured by Bradford assay. The lysates were mixed with 4× NuPage LDS Sample buffer supplemented with 200 mM DTT before denaturation at 70°C for 3 min. Typically, 5–15 µg total protein were resolved by SDS-PAGE on 4%–15% TGX Precast gels using 1× Laemmli running buffer (25 mM Tris, 192 mM glycine, 0.1%SDS pH 8.3). The proteins were transferred onto PVDF or nitrocellulose membranes using the Trans-Blot Turbo Transfer System (Bio-Rad) and transfer efficiency assessed by Ponceau Red staining. The membranes were blocked in PBS-T 5% milk (1× PBS, 0.1% Tween 20, 5% w/v milk powder) for 1 h at room temperature. Primary antibodies were diluted in PBS-T 5% milk and added to the membrane overnight at 4°C under constant shaking. After three PBS-T (1× PBS, 0.1% Tween 20) washes, each for 5 min, the membrane was incubated with HRP-conjugated secondary antibody in PBS-T 5% milk for 1 h at room temperature. Following three PBS-T washes, the western blots were developed using ECL on a Bio-Rad ChemiDoc MP Imaging System with auto-capture function.

### Northern blot

Typically, 15–20 µg of total RNA were mixed with 2× RNA gel loading dye (95% formamide; 0.025% xylene cyanol and bromophenol blue; 18 mM EDTA; 0.025% SDS), denatured for 5 min at 95°C, loaded onto a denaturing 8% polyacrylamide gel (8% Acrylamide/Bis 19:1, 6 M Urea, 0.5× TBE), and separated for 1–2 h at 350 V in 0.5× TBE. A semidry blotting apparatus was used to transfer the RNA onto a Hybond N^+^ membrane with 0.8 mA/cm^2^ for 2.5 h in 0.5× TBE. Following, RNA and membrane were crosslinked by UV exposure at 150 mJ/cm^2^.

The membrane was prehybridized (5× SSC, 7% SDS, 20 mM NaPi, 1× Denhardt solution, 0.1 mg/mL salmon sperm DNA) for 1 h at 50°C before the addition of a radioactively ^32^P-labeled DNA antisense oligonucleotide probe for overnight incubation at 50°C (Supplemental Table 2). The DNA probe was labeled with PNK and purified over a Chroma Spin chromatography column (Takara; Cat#636066) according to the manufacturer's guidelines. Finally, the membrane was washed three times with a high stringency buffer (5× SSC, 2% SDS) and three times with a low stringency buffer (1× SSC, 1% SDS), before visualizing the radioactive signal by 4–16 h exposure to a phosphorimaging screen.

### Protein expression and purification of recombinant full-length MBP-p62 and mutants thereof

Protein expression was performed according to Tarafder et al. (2019).

### Protein expression and purification of recombinant p62 (1–122, D69A/D71A/D73) for structural studies

The oligomerization deficient p62 truncation p62(1–122, D69A/D71A/D73A) was cloned into pCoofy4 (Ref: PMID: 23410102) using restriction-free cloning (van den Ent and Löwe 2006), transformed into the Rosetta 2(DE3) strain, and expressed in M9 medium with ^15^NH_4_Cl as the sole nitrogen source at 22°C O/N upon induction with 0.5 mM IPTG. The cells were lysed in a buffer containing 50 mM Tris pH 8, 750 mM NaCl, 10% glycerol, 20 mM imidazole, and cOmplete, EDTA-free Protease Inhibitor Cocktail using a microfluidizer (M-110L, Microfluidics Inc.). Subsequently, the protein was purified from the cleared lysate by Nickel affinity chromatography (HisTrap, Cytiva) in the same buffer. The affinity tag was removed by 3C protease cleavage (EMBL, PepCore), dialysis, and an additional passage over the HisTrap column. As a final purification step, oligomers were removed by gel filtration on a Superdex S75 10/300 in a buffer containing 20 mM MES pH 6.5, 100 mM NaCl, 0.2 mM TCEP, and 0.05% NaN_3_.

### Nuclear magnetic resonance spectroscopy

NMR spectra were acquired on a Bruker Avance III spectrometer with a cryogenic triple-resonance probe and a field strength of 18.8 T, corresponding to a proton Larmor frequency of 800 MHz at 298 K. For NMR titrations, protein samples at 80 µM concentration in 20 mM MES, pH 6.5, 100 mM NaCl, 0.2 mM TCEP, 0.05% NaN_3_ were titrated with full-length vault RNA1–1 in the same buffer. At each titration step, an apodization-weighted sampled HSQC was collected (Simon and Köstler 2019). Spectra were processed using NMRPipe (Delaglio et al. 1995) and analyzed using NMRFAM SPARKY (Lee et al. 2015). The yields and solubility of this construct did not enable sufficient signal-to-noise ratio for backbone assignments and subsequent mapping of chemical shift perturbations onto the structure.

### In vitro transcription of RNA

For biochemical assays: pUC57-T7-vaultRNA1–1 plasmids were linearized and used for in vitro transcription of RNA using the MEGAshortscript Kit (AM1354, Thermo Fisher) with ^32^P-αUTP (SRP-210, Hartmann) according to the manufacturer's guidelines. RNA was gel purified and phenol-chloroform extracted, dissolved in water and its concentration measured by QuBit assay (Thermo).

For NMR: pUC57-T7-vaultRNA1–1 plasmids were linearized and used for in vitro transcription of RNA in a large-scale reaction containing 100 mM HEPES-KOH pH 7.5, 10 mM MgCl_2_, 2 mM Spermidine-HCl, 40 mM DTT, 0.1 mg/mL BSA, 7.5 mM each NTP, 800 units/mL RNasin, 10 units/mL IPP, 25 µg/mL template DNA, and 10,000 units/mL T7 RNA polymerase and incubated 8 h at 37°C. The RNA was gel purified, extracted by electrophoresis, precipitated, and its concentration measured by NanoDrop.

### Electromobility shift assay (EMSA)

Before the reaction, RNA was denatured for 2 min at 95°C and subsequently refolded in the presence of 2.5 mM MgCl_2_. The EMSA reactions typically contained 10 nM–2 mM protein, 10 nM radioactively labeled RNA (150 fmol, 3 kcpm), 150 nM nonlabeled bacterial tRNA, 1 mg/mL of BSA, 10 mg/mL RNasin, 5 mM DTT, 0.5 mM PMSF, 2.5 mM MgCl_2_, 100 mM KCl; 20 mM HEPES pH7.9; 0.2 mM EDTA, and 20% glycerol. In the case of competitive EMSAs, the binding reaction was competed with nonlabeled RNA at a typical range of 0.1 to 2 µM that was added to the protein together with the labeled probe. Reactions were incubated for 10 min at room temperature, loaded onto a native 5% acrylamide gel, and run overnight at 70 V in 0.5× TBE. Subsequently, the gel was dried for 1 h at 80°C and signal visualized by exposure to a phosphorimaging screen.

### RNase footprinting

In vitro transcribed RNA was dephosphorylated with Fast Alkaline Phosphatase (FastAP) according to the manufacturer's guidelines and purified over a Zymo Quick-RNA Miniprep column. Subsequently, the RNA was radioactively labeled in a T4 PNK reaction with ^32^P-γUTP (Hartmann) and purified over a Chroma Spin chromatography column. Prior to RNase footprinting, the RNA was denatured for 10 min at 65°C and allowed to refold by ramping down 1°C/30 sec to room temperature. Per condition, 8 µL containing 20 µM 3′-end ^32^P-labeled RNA (∼1600 cps) and 6 µM protein were assembled in binding buffer [20 mM HEPES pH8, 100 mM KCl, 0.01% NP-40, 5% Glycerol, 2.5 mM MgCl_2_, 1 mM DTT supplemented with cOmplete Proteinase Inhibitor (EDTA-free)], and incubated for 10 min at room temperature. RNase A (5 ng/µL) was prediluted (1:200,000, 1:500,000) in H_2_O and 2 µL dilution added per reaction. The digest was incubated for 15 min at room temperature. Following, the RNA was extracted by Phenol-Chloroform extraction using TRI Reagent and dissolved in 2× RNA gel loading dye. For the alkaline ladder, 2 µL containing 20 µM 3′-end ^32^P-labeled RNA (∼400 cps) were mixed with 8 µL 50 mM NaHCO_3_ pH 9.2, incubated at 95°C for 5 min and supplemented with RNA gel loading dye. The samples and ladder were loaded onto a denaturing 8% polyacrylamide gel (8% acrylamide/bis 19:1, 6M Urea, 0.5× TBE) and separated at 42 W (50°C–55°C) in 0.5× TBE. The gel was fixed for 10 min in a bath of 6% acetic acid and 10% EtOH, dried on Whatman filter paper in a vacuum gel dryer at 80°C for 40 min, and the radioactive signal visualized by exposure to a phosphorimaging screen.

### Chemical structure probing of RNA

Per compound, four probing reactions were performed with one untreated control and three treated samples of varying concentrations. Therefore, 20 pmol in vitro transcribed RNA were diluted in 12 µL H_2_O, denatured at 95°C for 2 min and directly transferred on ice. The RNA was allowed to fold for 20 min at 37°C upon addition of 12 µL 2× folding buffer (40 mM HEPES pH8, 300 mM KCl, 0.02% NP-40, 10% glycerol, 5 mM MgCl_2_). Subsequently, 12 µL protein (15 µM) in 1× folding buffer was added to the reaction and incubated for 10 min at 37°C before splitting the reaction into 4 × 8 µL each. For the modification reactions, 92 µL Mod. buffer A for DMS (20 mM HEPES pH7, 150 mM KCl, 0.01% NP-40, 5% Glycerol, 2.5 mM MgCl_2_) or Mod. buffer B for CMCT (20 mM HEPES pH8, 150 mM KCl, 0.01% NP-40, 5% Glycerol, 2.5 mM MgCl_2_) were added. For DMS treatments 1 µL of 16×, 4×, or 2× DMS diluted in EtOH was added to the reactions, immediately mixed and incubated for 15 min at 37°C. For CMCT treatments, 5, 10, or 15 µL of CMCT solution (42 mg/mL in H_2_O) were added to the reactions, immediately mixed and incubated for 15 min at 37°C. The reactions were stopped on ice, RNA extracted with TRI reagent according to the manufacturer's guidelines and EtOH precipitated. Modified residues were detected by primer extension reaction using a radiolabeled DNA oligonucleotide (T4 PNK reaction and purification over a Chroma Spin 10 chromatography column) complementary to the 3′-end of the RNA. For primer extension, 1 µL of 10 µM radiolabeled primer was annealed to 2 µL of extracted RNA and used in a 5 µL reaction with AMV reverse transcriptase according to the manufacturer's guidelines. For the sequencing lanes, in vitro transcribed RNA was used in primer extension reactions that contained respective ddNTPs. The reactions were stopped with 20 µL Stop buffer (50 mM Tris pH8.3, 0.5% SDS, 7.5 mM EDTA) and the cDNA EtOH-precipitated and separated on a denaturing 8% polyacrylamide gel (8% acrylamide/bis 19:1, 8 M urea, 0.5× TBE) at 42W in 0.5× TBE. Subsequently, the gel was fixed for 10 min in a bath of 6% acetic acid and 10% EtOH, dried on Whatman filter paper in a vacuum gel dryer at 80°C for 40 min, and the radioactive signal visualized through exposure to a phosphorimaging screen.

### Reverse transcription and quantitative PCR (RT-qPCR)

Reverse transcription was performed with 500 ng of total RNA or 7 µL of purified RNA from IPs in a 10 µL reaction using the Maxima First Strand Synthesis kit (Thermo; Cat#K1641) according to the manufacturer's guidelines. Control reactions that did not include the reverse transcriptase (-RT) were included for each sample. The obtained cDNA was diluted 1:20–1:50 in nuclease-free water and assembled in 10 µL qPCR reactions with 5 µL cDNA, 4.6 µL Fast SB SYBR Green Master Mix, 0.2 µL each primer (10 µM). Primer sequences can be found in Supplemental Table 2. The analysis was performed on an Applied Biosystems QuantStudio 6 Flex Real-Time PCR system in the default standard two-step cycling run. Relative expressions were calculated with the ΔΔCT method. The mean *C*_t_ value was used when normalizing to multiple housekeeping genes.

### Quantification and statistical analysis

Statistical analysis was performed with GraphPad Prism 8.3.1 (279; www.graphpad.com). Details for each experiment can be found in the corresponding figure legend. Image analysis was performed with Fiji Image J 2.0.0 (Schindelin et al. 2012, https://imagej.net/Fiji). Structure representations were generated using PyMOL Molecular Graphics System 2.0.7 (https://pymol.org). NMR analysis was performed with NMRPipe (Delaglio et al. 1995, https://www.ibbr.umd.edu/nmrpipe/) and NMRFAM SPARKY (Lee et al. 2015; https://nmrfam.wisc.edu/nmrfam-sparky-distribution/).

## SUPPLEMENTAL MATERIAL

Supplemental material is available for this article.

## Supplementary Material

Supplemental Material

## Figures and Tables

**Magdalena Büscher:**
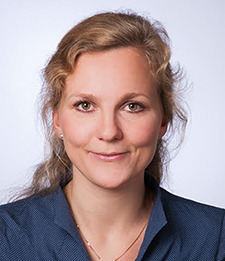


## MEET THE FIRST AUTHOR

***Meet the First Author(s)* is a new editorial feature within *RNA*, in which the first author(s) of research-based papers in each issue have the opportunity to introduce themselves and their work to readers of *RNA* and the RNA research community. Magdalena Büscher is the first author of this paper, “Vault RNA1–1 riboregulates the autophagic function of p62 by binding to lysine 7 and arginine 21, both of which are critical for p62 oligomerization.” Magdalena completed her joint PhD degree between the European Molecular Biology Laboratory (EMBL) and the University of Heidelberg in the laboratory of Prof. Dr. Matthias W. Hentze in 2021. During her time as a predoctoral fellow at EMBL, she focused on the molecular mechanisms of p62-riboregulation via the small noncoding vault RNA1–1.**

**What are the major results described in your paper and how do they impact this branch of the field?**

Riboregulation of p62-dependent autophagy by the small noncoding vault RNA1–1 is a prime example of how an RNA can directly control the function of a protein, just like protein–protein interactions and PTMs. The detailed molecular study presented here uncovers how binding of vault RNA1–1 interferes with PB1 domain-mediated oligomerization of p62, and therefore describes an underlying principle that could well apply to other potential riboregulators.

Our systematic approach maps the binding interface from the protein and RNA side and highlights the prominent role of a central flexible loop region of vault RNA1–1 in riboregulation. This loop region distinguishes vault RNA1–1 from the other human vault RNA paralogues and is therefore suggested to be a determinant of specificity.

**What led you to study RNA or this aspect of RNA science?**

We were intrigued by the fact, that RNA could play a far more “active” role in directly regulating key cellular processes. The discovery that hundreds of well-characterized cellular proteins associate with RNA led us to follow up on this fascinating aspect of RNA science.

**During the course of these experiments, were there any surprising results or particular difficulties that altered your thinking and subsequent focus?**

The finding that mutations within the ZZ domain of p62 indirectly affect RNA binding were indeed puzzling at first. We are happy that our systematic approach uncovered the distinct roles that the different domains of p62 have in vault RNA1–1 binding and regulation of the RNA–protein interaction.

**If you were able to give one piece of advice to your younger self, what would that be?**

You have to be open-minded to see new opportunities, but brave enough to seize them.

**Are there specific individuals or groups who have influenced your philosophy or approach to science?**

I loved the vibrant scientific discussions, inspiring interdisciplinary approaches and cultural diversity at EMBL. The open-minded, “everything is possible” attitude and top-class collaborations that you can find there at a minute walking distance make this place a truly unique scientific hub. I would like to thank everyone who is (and was) part of EMBL for this great experience!
