# Supplementary material for: Vault RNA1–1 riboregulates the autophagic function of p62 by binding to lysine 7 and arginine 21, both of which are critical for p62 oligomerization
Source: RNA. 2022 May;28(5):742–55. doi: 10.1261/rna.079129.122 (PMC9014876; doi:10.1261/rna.079129.122)
Supplement: Supplemental Material [file supp_28_5_742__DC1.html]

Vault RNA1-1 riboregulates the autophagic function of p62 by binding to K7/R21 that are critical for p62 oligomerisation — Vault RNA1–1 riboregulates the autophagic function of p62 by binding to lysine 7 and arginine 21, both of which are critical for p62 oligomerization — Supplemental Material 

# Vault RNA1–1 riboregulates the autophagic function of p62 by binding to lysine 7 and arginine 21, both of which are critical for p62 oligomerization

## Supplemental Material

- Supplemental\_Figures\_and\_Tables.docx
